# Contributions and Prognostic Values of N6-Methyladenosine RNA Methylation Regulators in Hepatocellular Carcinoma

**DOI:** 10.3389/fgene.2020.614566

**Published:** 2021-01-15

**Authors:** Li-Wen Qi, Jian-Hui Jia, Chen-Hao Jiang, Jian-Ming Hu

**Affiliations:** ^1^Department of Clinical Oncology, Liaoning Cancer Hospital, Graduate School of Dalian Medical University, Dalian, China; ^2^Department of Pathology, The First Affiliated Hospital, Shihezi University School of Medicine, Shihezi, China; ^3^Department of Gastrointestinal Tumor, Liaoning Cancer Hospital, Cancer Hospital of China Medical University, Shenyang, China

**Keywords:** consensus clustering, gene signature, hepatocellular carcinoma, TCGA, UCSC, METTL3, YTHDF1

## Abstract

**Introduction:**

The methylation at position N6 of adenine is called N6-methyladenosine (m6A). This transcriptional RNA modification exerts a very active and important role in RNA metabolism and in other biological processes. However, the activities of m6A associated with malignant liver hepatocellular carcinoma (LIHC) are unknown and are worthy of study.

**Materials and Methods:**

Using the data of University of California, Santa Cruz (UCSC), the expression of M6A methylation regulators in pan-cancer was evaluated as a screening approach to identify the association of M6A gene expression and 18 cancer types, with a specific focus on LIHC. LIHC datasets of The Cancer Genome Atlas (TCGA) were used to explore the expression of M6A methylation regulators and their clinical significance. Gene Ontology (GO) analysis and Gene Set Enrichment Analysis (GSEA) were used to explore the underlying mechanism based on the evaluation of aberrant expression of m6A methylation regulators.

**Results:**

The expression alterations of m6A-related genes varied across cancer types. In LIHC, we found that in univariate Cox regression analysis, up-regulated m6A modification regulators were associated with worse prognosis, except for ZC3H13. Kaplan–Meier survival curve analysis indicated that higher expression of methyltransferase-like protein 3 (METTL3) and YTH N6-methyladenosine RNA binding protein 1 (YTHDF1) genes related to the worse survival rate defined by disease-related survival (DSS), overall survival (OS), progression-free interval (PFI), and disease-free interval (DFI). Up-regulated m6A methylation regulator group (cluster2) obtained by consensus clustering was associated with poor prognosis. A six-gene prognostic signature established using the least absolute shrinkage and selection operator (LASSO) Cox regression algorithm performed better in the early (I + II; T1 + T2) stages than in the late (III + IV; T3 + T4) stages of LIHC. Using the gene signature, we constructed a risk score and found that it was an independent predictive factor for prognosis. Using GSEA, we identified processes involved in DNA damage repair and several biological processes associated with malignant tumors that were closely related to the high-risk group.

**Conclusion:**

In summary, our study identified several genes associated with m6A in LIHC, especially METTL3 and YTHDF1, and confirmed that a risk signature comprised of m6A-related genes was able to forecast prognosis.

## Introduction

RNAs such as long non-coding RNAs (lncRNAs), transfer RNAs (tRNAs), messenger RNAs (mRNAs), microRNAs (miRNAs), and ribosomal RNAs (rRNAs) have been reported to be subjected to over 100 types of chemical modifications (Saletore et al., 2012; Huang et al., 2020b). Among them, N6-methyladenosine (m6A) was first identified in 1974. M6A is a reversible post-transcriptional modification and is considered to be the most common methylation site of eukaryotic mRNAs (Desrosiers et al., 1974; Wei et al., 1975). M6A methyltransferases (also called “writers”) responsible for this type of RNA modification include KIAA1429, zinc finger CCCH domain-containing protein 13 (ZC3H13), methyltransferase-like protein 3 (METTL3), METTL14, METTL16, RNA-binding motif protein (RBM15), and Wilms Tumor 1-associated protein (WTAP) (Akichika et al., 2019). The α-ketoglutarate-dependent dioxygenase alkB homolog 5 (ALKBH5) and fat mass and obesity-associated protein (FTO) were called m6A demethylases (also called “erasers”) and detached m6A (Fu et al., 2013; Zheng et al., 2013). M6A-binding proteins (also called “readers”) include YTHDC1-2, insulin-like growth factor 2 mRNA-binding proteins (IGF2BPs), heterogeneous nuclear ribonucleoproteins (HNRNPs), and the YTH N6-methyladenosine RNA binding proteins 1 to 3 (YTHDF1–3) (Wang et al., 2014; Liao et al., 2018).

Except its effects on the synthesis/metabolism of RNA(Roignant and Soller, 2017), effects on the immune response, metabolism, viralreplication, cancer development, embryogenesis, and other biologicalprocesses have been found to be associated with modification of m6A(Muthusamy, 2020). Somestudies have shown that aberrant m6A modification may act to induceor inhibit cancer progression in malignant tumors (Chen X. Y. et al., 2019; Sun et al., 2019), as in, for example, the hematological malignancyacute myeloid leukemia (AML). Some studies (Kwok et al., 2017) haverevealed that alterations in m6A regulatory genes confer a worsesurvival. As oncogenes of AML, METTL3 (Vu et al., 2017), METTL14 (Weng et al., 2018), WTAP (Bansal et al., 2014), FTO (Li et al., 2017), YTHDF2 (Li Z. et al., 2018), and IGF2BP1 (Zhou et al., 2017) participate in tumor processes through a variety of pathways, including the promotion of the growth of cancer cells and inhibition of apoptosis. In urological tumors, such as prostate cancer (PCA), studies (Ji et al., 2020) have shown that the expression of IGF2BP3, HnRNPA2B1, METTL14, and ALKBH5 was associated with recurrence-free survival. METTL3 (Cai et al., 2019) and YTHDF2 (Li J. et al., 2018) as oncogenes promoted PCA cell proliferation and migration. In neoplasms of the skin, such as uveal melanoma (UM), higher expression of ALKBH5, KIA1429, and YTHDF1 was found to be associated with worse prognosis (Tang et al., 2020). Conversely, some m6A genes have been found to act as tumor suppressor genes or oncogenes, for example, METTL3 and METTL14 (Cui et al., 2017), in neurological tumors such as glioblastoma (GBM). In addition, a number of other tumors have also been found to be associated with M6A methylation regulators (Zhao et al., 2020).

Therefore, the identification of changes in m6A expression in pan-cancer was identified as the starting point of the study. Among these neoplasms, m6A in LIHC stood out because of its close relationship with prognosis, and thus, it became the main focus of our study. LIHC ranks sixth among global cancer incidence and ranks fourth in cancer-related deaths (Bray et al., 2018). The prognosis of LIHC is unsatisfactory because of the easy recurrence of the tumor after treatment (Llovet et al., 2016). Thus, there is a need to identify prognostic markers able to improve the therapeutic effects. In previous studies on LIHC, some scholars found that the relationship between M6A methylation regulators and prognosis is not clear and controversial. For example, METTL14 plays an oncogenic role in LIHC (Lin et al., 2016), while Ma et al. (2017) have proved that METTL14 is an anti-metastatic factor. Similarly, some studies have shown that YTHDF2 inhibits the development of LIHC (Zhong et al., 2019), but others have found that the overexpression of YTHDF2 is related to the poor prognosis of LIHC (Chen et al., 2018). In addition, because of the interaction between M6A-related genes, there is still no criterion for whether the combination of these genes can better predict the prognosis of patients. To gain a better comprehensive and accurate understanding of m6A methylation regulators in LIHC with prognosis, we did this research.

## Materials and Methods

### Cancer Datasets

All pan-cancerous gene expression datasets (RNA-seq) and survival information were obtained from The Cancer Genome Atlas (TCGA^1^). UCSC Xena is a database maintained by the University of California, Santa Cruz. It contains public datasets including TCGA, ICGC, TARGET, and other databases and standardizes the data to make it easier for follow-up analysis (Cline et al., 2013). We analyzed 33 different TCGA projects, each project represented a specific cancer type, including kidney renal papillary cell carcinoma (KIRP); kidney chromophobe (KIC); brain lower grade glioma (LGG); stomach adenocarcinoma (STAD); breast cancer (BRCA); lung adenocarcinoma (LUAD); rectum adenocarcinoma (READ); colon adenocarcinoma (COAD); acute myeloid leukemia (AML); testicular germ cell tumors (TGCT); liver hepatocellular carcinoma (LIHC); uterine carcinosarcoma (UCS); ovarian serous cystadenocarcinoma (OV); head and neck squamous carcinoma (HNSC); lung squamous cell carcinoma (LUSC); thyroid carcinoma (THCA); lymphoid neoplasm diffuse large b-cell lymphoma (DLBC); prostate adenocarcinoma (PRAD); skin cutaneous melanoma (SKCM); bladder urothelial carcinoma (BLCA); uterine corpus endometrial carcinoma (UCEC); glioblastoma multiforme (GBM); cervical squamous cell carcinoma and endocervical adenocarcinoma (CESC); adrenocortical carcinoma (ACC); sarcoma (SARC); pancreatic adenocarcinoma (PAAD); pheochromocytoma and paraganglioma (PCPG); esophageal carcinoma (ESCA); thymoma (THYM); mesothelioma (MESO); kidney renal clear cell carcinoma (KIRC); uveal melanoma (UVM); and cholangiocarcinoma (CHOL). In order to use the most recent data available, liver cancer data in the final study were obtained from TCGA^2^ database, including gene expression datasets (RNA-seq) belonging to 374 patients with liver cancer and 50 normal controls, as well as the clinical and pathological data in the database.

### Screening of M6A Methylation Regulatory Genes

We identified 21 m6A regulators from recently published review papers in PubMed (Han and Choe, 2020; Wang T. et al., 2020; Zhao et al., 2020), including eleven reader, eight writer, and two eraser genes. Among these, 20 m6A methylation regulators were selected for this study, and all were present in the gene expression datasets (RNA-seq).

### Bioinformatics Analysis

All data analysis was based on R (v3.4.1). In the first step, all samples were analyzed for differential expression of m6A in different normal and tumor tissues, excluding tumor types in which the normal group had less than five samples. Wilcoxon’s rank sum test was used to find the divergence across m6A genes. The identification criteria for m6A having differential expression for each tumor type was a *p*-value < 0.05 and at least a two-fold change in expression. A statistical analysis was needed to evaluate the relationship between survival and M6A-related genes in tumors, univariate Cox regression analysis. The risk or protective genes correspond to the hazard ratio of (HR) > 1 or < 1, respectively. The relevance with m6A-related genes and disease-related survival (DSS), overall survival (OS), disease-free interval (DFI), and progression-free interval (PFI) was shown by Kaplan–Meier survival curves, obtained by the “survival” and “survminer” R package. In survival analysis, genes are ranked from high to low in terms of expression level, and the “median” of the expression level is used as the cutoff value; higher than this value is considered as high expression, and lower than this value is considered as low expression.

Liver hepatocellular carcinoma samples were grouped by category using “ConsensusClusterPlus”(Wilkerson and Hayes, 2010), and the number of groups was denoted by “*k*.” The LIHC datasets were grouped into distinct and non-overlapping groups according to the consistent expression of m6A genes. An optimal prediction model for determination of prognosis was constructed using the least absolute shrinkage and selection operator (LASSO) Cox regression algorithm (Sauerbrei et al., 2007). According to the cutoff value, patients were grouped into high-risk and low-risk groups based on the risk score (using the risk signature). Using the GLMNET package in R to perform the LASSO regression, the risk score = $\sum_{j=1}^{n}{\mathrm{Coefj}}^{*}\,\mathrm{xj}$. In the formula, Coefj and *xj*, respectively, symbolize the coefficient and the *z*-score-normalized related expression levels of each gene. Genes with a high correlation were chosen and shrunk to prevent over-fitting, and factors with fairly low association with were removed from the model (Sauerbrei et al., 2007). Finally, an optimal prognostic model was constructed using m6A regulatory genes, and each patient received a risk score. In the display of the heat map, each small square represents each gene, and the color represents the expression level of each gene. The greater the expression level, the darker the color (red up-regulation and green down-regulation).

Gene Ontology (GO) analysis was used to explore differences in biological pathways. Background data of R software “org.Hs. eg.db” was used to obtain the gene ID (Entrez gene ID) of potential targets, and then, the package (Bioconductor) was used to analyze the GO function enrichment of these potential targets. The correlation with m6A-related genes was determined by Spearman’s correlation. The string database^3^ is used to generate the interactive network of these genes (Szklarczyk et al., 2017). Next, the biological process was divided by the risk score of the high- and the low-risk group, and Gene Set Enrichment Analysis (GSEA) was used in the LIHC cohort. KEGG gene sets, phenotypic tags (high) and (low) expression files were loaded into GSEA (v4.0.3; Broad Institute, Cambridge, MA) software and the permutation test was run 1,000 times.

### Statistics

The differences in continuous variables and categorical variables among different groups were compared by means of the Student’s *t*-test and the χ^2^ test. The differences in survival rates between groups were derived from the Kaplan–Meier survival curve and were verified by the two-sided logrank test. The prognostic capability of the resulting risk score was assessed by singular and multiple Cox regression analysis. A *p*-value < 0.05 was considered to be statistically significant. R (v3.4.1^4^) was used for all statistical analysis.

## Results

### M6A-Related Gene Expression

**FIGURE 1:**
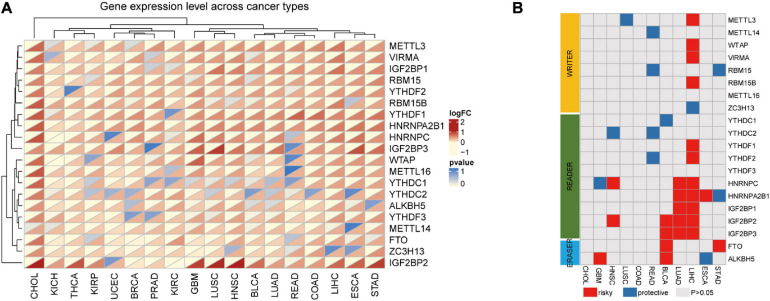
The expression of m6A-related genes in the pan-cancer analysis. **(A)** Gene expression variation of m6A genes across 18 cancer types. The heatmap shows the fold changes. Red represents overexpression genes and yellow represents lower expression genes. *P* < 0.05 was considered as statistically significant. **(B)** The relationship between higher expression of m6A-associated genes and patient survival, with red and blue representing worse and better survival, respectively. Only *P*-values < 0.05 are shown.

In TCGA, we selected the RNA expression datasets relative to 20 m6A-related genes in our study (“writers”: METTL3, METTL14, METTL16, WTAP, KIAA1429, RBM15, RBM15B, and ZC3H13; “readers”: YTHDC1, YTHDC2, YTHDF1, YTHDF2, YTHDF3, HNRNPC, HNRNPA2B1, IGF2BP1, IGF2BP2, and IGF2BP3; and “erasers”: FTO and ALKBH5). The expression changes of m6A-related genes are shown as heat maps, with red and yellow representing up-regulated and down-regulated genes, respectively. In the pan-cancer data of 33 cancer types, excluding the tumor types whose normal samples were less than 5, a total of 18 kinds of tumors were clustered into two categories according to the dysregulated expression of m6A-related genes. The first seven were mainly genitourinary system tumors, such as KIRC, UCEC, BRCA, PRAD, KICH, and KIRP. The remaining 11 were mainly respiratory, digestive, and head and neck system tumors and included CHOL, ESCA, STAD, LIHC, COAD, READ, LUSC, LUAD, HNSC, and GBM. Compared with the first seven tumors, the 20 m6A-related genes in the latter 11 tumors were mainly up-regulated (METTL3, VIRMA, RBM15, RBM15B, YTHDF1, YTHDF2, IGF2BPs, and HnRNP family) and were mainly “writers” and “readers.” However, although METTL14 and ZC3H13 are “writer” genes, they were down-regulated in the first seven kinds of tumors listed above. Similarly, an up-regulated gene expression was found in upper digestive system tumors including ESCA, STAD, HIHC, and CHOL. In addition, we found that IGF2BP3 was up-regulated in 17 tumors except for THCA (Figure 1A). Based on these findings, we preliminarily found that changes in the expression of 20 m6A-related genes may vary across cancer types. These results also reveal the highly heterogeneous expression changes of m6A in different cancer types, suggesting that the dysregulation of m6A regulatory factors may play an important role in different cancer environments.

### The Prognostic Role of m6A

Alterations in m6A are prevalent in 18 types of cancers. The relationship between the 20 m6A genes and survival time in patients in the latter 11 tumor types was assessed by univariate Cox analysis. Genes were mainly up-regulated. HR > 1 or HR < 1 corresponded to damaging or protective genes, respectively. We found that the tumor survival rates studied were all related to at least one of the m6A methylation regulators.

Some m6A methylation regulators were considered to be risk genes, such as the insulin-like growth factor 2 mRNA-binding proteins (IGF2BPs), including IGF2BP1, IGF2BP2, and IGF2BP3. Poor survival rates in patients were associated with the increased expression of these genes across cancer types, such as IGF2BP1 (HR = 1.324527, *P* = 2.38E-06) in LUAD and IGF2BP2 (HR = 1.198617, *P* = 0.006603) and IGF2BP3 (HR = 1.570331, *P* = 0.000198) in LIHC. In contrast, we found that several m6A regulators were protective genes for tumors, such as in READ, where the high expression of m6A regulators YTHDF2, YTHDC2, RBM15, and METTL14 was significantly correlated with better survival (Figure 1B). Among these, we found that m6A in LIHC was associated with the largest number of genes associated with survival, including METTL3, WTAP, KIAA1429, RBM15, ZC3H13, YTHDF1, YTHDF2, HNRNPC, HNRNPA2B1, IGF2BP1, IGF2BP2, and IGF2BP3. Except for ZC3H13, the high expression of these genes was related to the worse survival rate of LIHC; thus, we focused on LIHC in this study.

**FIGURE 2:**
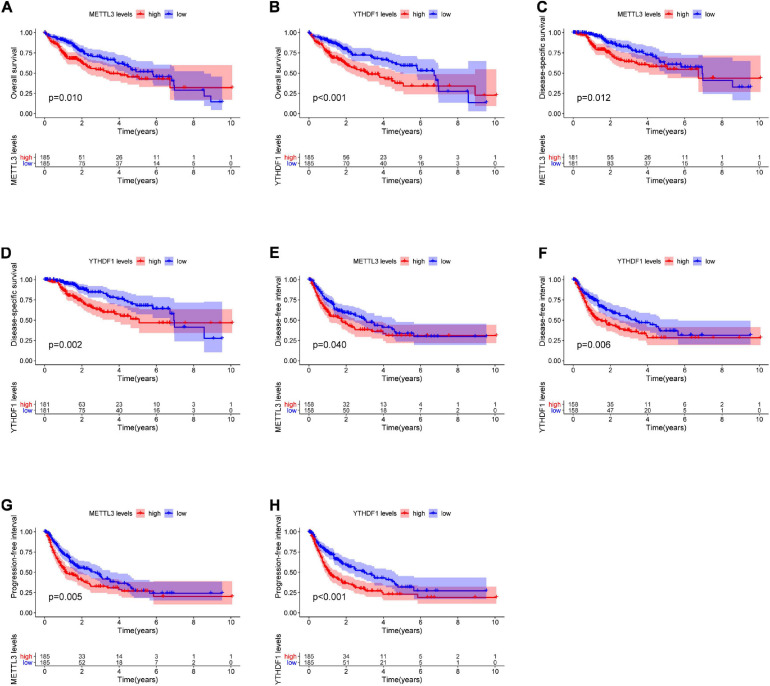
The Kaplan–Meier curves for OS, DSS, DFI, and PFI in LIHC. Kaplan–Meier survival curves showing differences in OS **(A,B)** and DSS **(C,D)** stratified according to METTL3 and YTHDF1 expression. Kaplan–Meier survival curves showing difference in DFI **(E,F)** and PFI **(G,H)** stratified according to METTL3 and YTHDF1 expression. LIHC, liver hepatocellular carcinoma; OS, overall survival; DSS, disease-associated survival; DFI, disease-free interval; PFI, progression-free interval.

The relationship between the m6A regulators in LIHC and PFI, DSS, OS, and DFI was determined by the Kaplan–Meier method. The OS of patients with higher expression of METTL3, YTHDF2, YTHDF1, IGF2BP3, RBM15B, RBM15, and HNRNPA2B1 was worse than the low-expression group. The DSS of patients with higher expression of METTL3, YTHDF1, METTL16, HNRNPC, and RBM15 was significantly poorer than that of patients with low expression. The DFI of patients with higher gene expression of METTL3, YTHDF1, HNRNPC, and HNRNPA2B1 was significantly lower than that in the low-expression group. The PFI of patients with higher gene expression of METTL3, YTHDF1, WTAP, IGF2BP3, YTHDC1, RBM15B, RBM15, and HNRNPA2B1 was worse than the low-expression genes. Overall, we found that the combined higher expression of METTL3 and YTHDF1 correlated with worse prognosis of patients in terms of OS, DSS, DFI, and PFI (Figure 2).

### Subgroup Identification Based on Consensus Clustering

**FIGURE 3:**
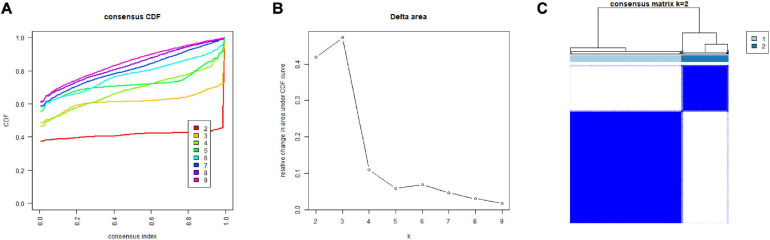
Consensus clustering of m6A-related genes. **(A)** Cumulative distribution function (CDF) for LIHC. **(B)** The area under the CDF curve in LIHC. **(C)** Consensus clustering matrix for LIHC. LIHC, liver hepatocellular carcinoma.

In order to deeper investigate the clinical correlation of 20 m6A related-genes in LIHC, we used the class discovery tool “ConsensusClusterPlus” to group LIHC samples according to gene expression patterns and used “*k*” to indicate the number of subgroups. The cumulative distribution function (CDF) graph (Figure 3A) shows the cumulative distribution function when “*k*” takes different values (*k* = 2–9). As shown in the figure, when *K* = 3, CDF is in a position of slow growth and the clustering analysis result is the most reliable at this time. The delta area plot (Figure 3B) shows the relative changes in the area under the CDF curve compared to *k* and *K* - 1. The first point represents the total area under the CDF curve at *K* = 2, not the relative change of the area, because there is no *K* = 1. When *k* = 4, the area under the curve increases only slightly, so 3 is the appropriate *k* value. Figure 3C shows the matrix heat map when *k* = 2: the rows and columns of the matrix represent samples; the values of the consistency matrix are represented by white to dark blue from 0 (impossible to cluster together) to 1 (always clustered together); and the consistency matrix is arranged according to the consistency classification (the tree above the heat map). The category is divided by the long bar between the tree and the heat map. According to the CDF and the delta area plot, we can temporarily consider grouping patients into three groups. However, the matrix heat map showed that when *k* = 3, the sample size of one of the groups was too small and the correlation between groups was high. In summary, in accordance with the m6A-related gene expression approach for consensus clustering, when *k* = 2, the LIHC cohort could be separated into two subgroups which were different and did not overlap.

**FIGURE 4:**
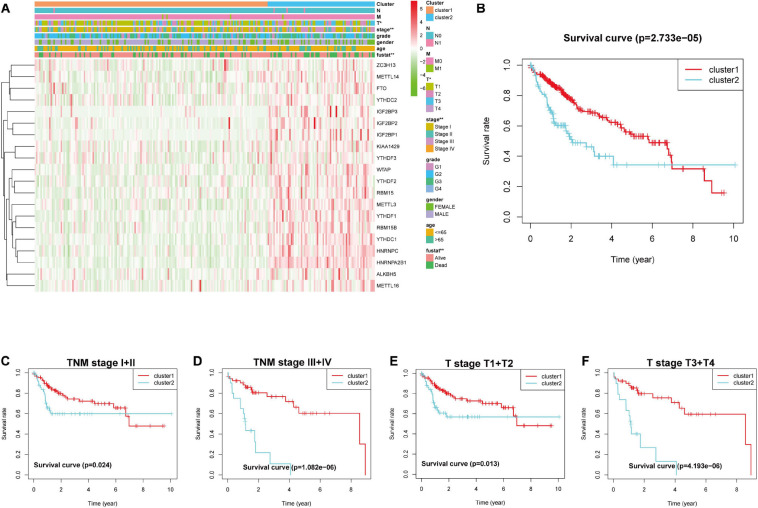
Heatmap and clinical features in **(A)** cluster1 and cluster2, stratified by the m6A-related gene consensus analysis. **(B)** Kaplan–Meier survival curves for groups of clusters in LIHC. Kaplan–Meier OS curves for patients with **(C)** stage I + II and **(D)** III + IV in LIHC. Kaplan–Meier OS curves for patients with **(E)** stageT1 + T2 and **(F)** T3 + T4 in LIHC. LIHC, liver hepatocellular carcinoma; OS, overall survival.

According to the heat map obtained relative to the gene expression characteristics of the two groups after observation consensus clustering, we found that the genes in the cluster2 group were generally highly expressed. Next, we investigated whether there was a distinction between the clinical and pathological features between the two groups. The outcomes showed obvious differences between tumor T stage and clinical stage (Figure 4A). According to the correlation between grouping and clinical data, we found that the OS of cluster1 (*n* = 261) group was better than the cluster2 group (*n* = 109) (Figure 4B). Next, we determined that the expression patterns of 20 m6ARNA methylation regulatory genes could predict clinical outcomes of LICH subgroups stratified by clinical stage. This consensus clustering based on expression pattern was capable of predicting prognosis in both early stage (I + II; T1 + T2) (Figures 4C,E) and late stage (III + IV; T3 + T4) (Figures 4D,F).

**FIGURE 5:**
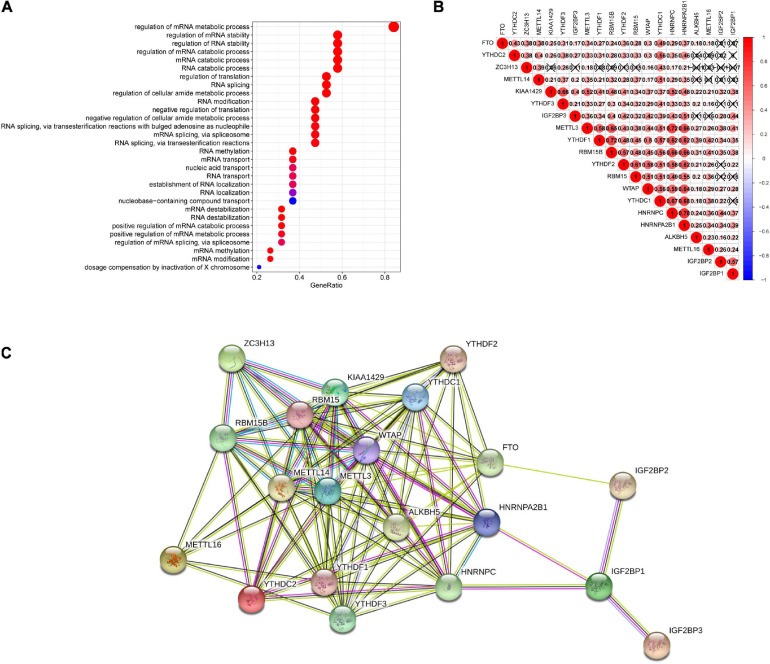
Spearman’s correlation analysis and functional annotations of 20 m6A-related genes. **(A)** GO annotation. **(B)** Spearman correlation analysis in 20 m6A modification regulators genes in LIHC. **(C)** String protein–protein interaction network. LIHC, liver hepatocellular carcinoma.

We further annotated these genes according to GO terms and discovered that they were mainly involved in mRNA processing-related pathways, containing RNA modification, regulation of RNA splicing, metabolism, transport, and stability, which were consistent with the RNA modification function of m6A (Figure 5A). In addition, m6A methylation regulators do not work in isolation. Previous evidence has shown that cooperation among writers, readers, and erasers is the background of carcinogenesis (Panneerdoss et al., 2018). Using Spearman’s correlation analysis to calculate the correlation of these genes in LIHC, we identified m6A-related genes in the same functional category showing highly interrelated expression patterns, which overlapped those of authors, erasers, and readers. For example, the expression levels of METTL3, YTHDF1, RBM15B, YTHDF2, RBM15, WTAP, YTHDC1, HNRNPC, HNRNPA2B1, and KIAA1429 genes closely associated with each other, while the expression level of ZC3H13 gene was weakly correlated or not associated with other genes except for METTL14 and YTHDC1 (Figure 5B). In addition, the interplay of these genes also existed in protein–protein interaction networks, especially in writers (Figure 5C).

### Building the Prognostic Signatures

Cox regression analysis and Kaplan–Meier survival curves were used in univariate analysis to determine the correlation between genes and prognosis, and consensus clustering was used to further explore clinical correlations. Through protein–protein interaction network analysis and Spearman’s correlation analysis, we found the functions of these m6A methylation regulators were not isolated, and there was cooperation among writers, erasers, and readers. Therefore, in order to improve the predictive ability of m6A in LICH, we used the LASSO Cox regression algorithm to eliminate genes that did not meet our requirements in order to establish suitable prognostic gene markers. This method allowed us to compute a patient’s risk score by combining the level of gene expression with the risk coefficient.

**FIGURE 6:**
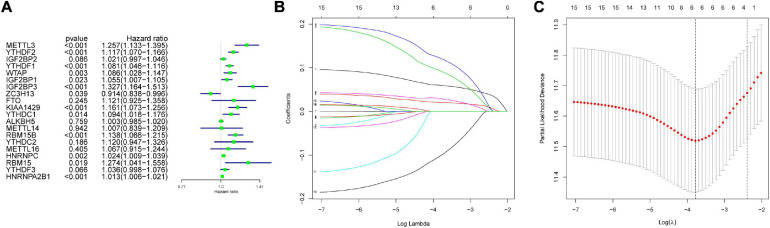
Screening of variables. **(A)** The hazard ratios (HR) and 95% confidence intervals (CI) of 20 m6A modification regulators in LIHC was computed by univariate Cox regression. In the 15 genes, high correlation genes were chosen and shrunk to prevent over-fitting **(B,C)** and finally produced a six-gene signature in LASSO regression. LIHC, liver hepatocellular carcinoma; LASSO, least absolute shrinkage and selection operator.

**FIGURE 7:**
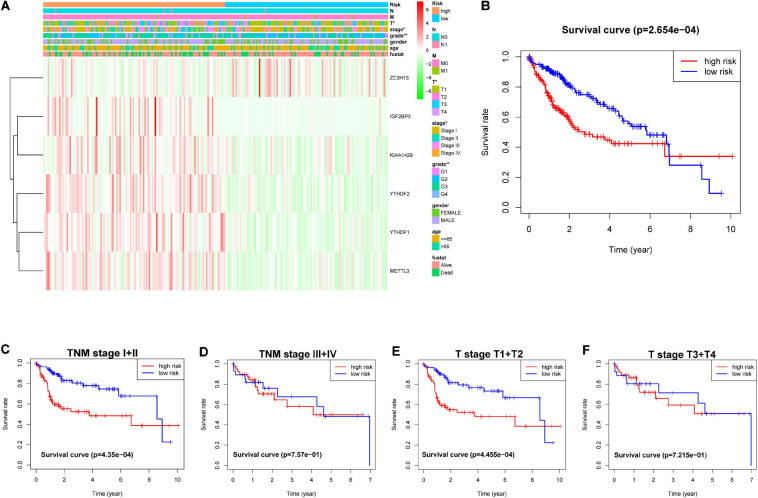
**(A)** Heatmap and clinicopathological characteristics of the subgroup classified by the six-gene prognostic signature in LIHC. **(B)** Kaplan–Meier survival curves of LIHC subgroups defined by the six-gene signature based on the LASSO regression. Kaplan–Meier OS curves for patients with **(C)** stage I + II and **(D)** III + IV LIHC. Kaplan–Meier OS curves for patients with stage **(E)** T1 + T2 and **(F)** T3 + T4 LIHC. LIHC, liver hepatocellular carcinoma; OS, overall survival.

**FIGURE 8:**
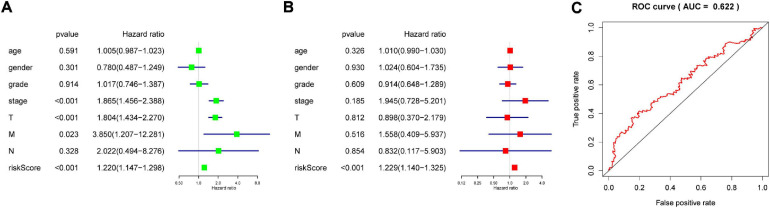
The role of risk score in prognosis. Single **(A)** and multiple **(B)** factors used in the Cox analysis to develop the risk scores in LIHC. **(C)** ROC curve showing the supporting the performance of the model. LIHC, liver hepatocellular carcinoma; ROC, receiver operating characteristic.

The genes analyzed by univariate Cox analysis were screened according to the standard of *P* < 0.1 by the LASSO Cox regression algorithm (Figure 6A). Fifteen genes that met the requirements were substituted into the model, and we chose and shrunk the genes with high correlation to prevent over-fitting (Figures 6B,C). As a result, LASSO regression produced a six-gene signature, including YTHDF2, YTHDF1, METTL3, IGF2BP3, KIAA1429, and ZC3H13. The resulting risk score divided the LIHC patients into the low- and high-risk groups of OS. We continued to observe whether there were differences in clinical and pathological features between the two groups. The results showed obvious differences in T stage, pathological stage, and clinical stage (Figure 7A). The OS in the low-risk (*n* = 185) group was significantly better than that in the high-risk group (*n* = 185) (Figure 7B). We also examined whether the high-risk or low-risk score could predict clinical outcome in LICH subgroup stratified by clinical stage. The results showed the gene expression in stages I + II out-performed prognosis prediction over gene expression in stages III + IV in LICH (Figures 7C,D), and was better in calculating prognosis for stages T1 + T2 than for stages T3 + T4 (Figures 7E,F). Next, we performed single and multiple Cox analysis. The resulting risk score was confirmed to be an independent prognostic factor for LIHC and showed good sensitivity and specificity, as demonstrated by the receiver operating characteristic (ROC) curves (Figure 8).

### Signal Pathways and Cellular Processes Related to M6A Regulators

**FIGURE 9:**
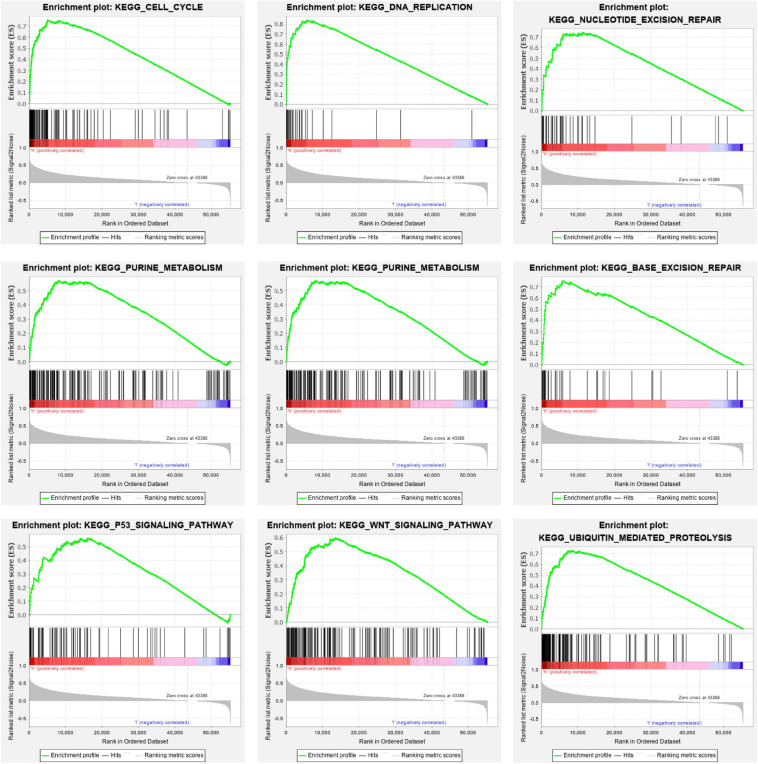
Cellular processes and pathways in LIHC subsets, defined by risk scores. GSEA showed that the low survival subgroup was obviously correlated with processes such as the cell cycle, DNA replication, WNT signal pathway, and protein degradation. GSEA, Gene Set Enrichment Analysis; LIHC, liver hepatocellular carcinoma.

In order to further investigate the effects of m6A regulators on signal pathways and cellular processes, we turned to GSEA to inspect the signal pathways involved in the high-risk and low-risk prognosis groups. We found that the high-risk group was characterized by the following biological processes/signal pathways. Cell cycle (Nes = 2.13, Fdr = 0.001), DNA replication (Nes = 1.97, *p* = 0.006), pyrimidine metabolism (Nes = 2.12, *p* = 0.000), nucleotide excision repair (NER) (Nes = 2.05, FDR = 0.001), base excision repair (BER) (Nes = 2.05, *p* = 0.000), WNT signaling pathway (Nes = 1.99, *p* = 0.000), purine metabolism (Nes = 2.08, *p* = 0.000), p53 signaling pathway (Nes = 1.88, *p* = 0.000), and ubiquitin-mediated proteolysis (Nes = 2.02, *p* = 0.000). The loss of control of the following processes correlated with oncogenesis: cell cycle regulation, WNT signaling pathway, p53 signaling pathway, and ubiquitin-mediated proteolysis (Figure 9).

## Discussion

M6A is the most universal chemical modification in RNA. Although previous studies have shown that m6A is involved in many biological processes (Han et al., 2019; Shi et al., 2019; Zhong et al., 2019), the role of m6A modification in cancer and clinical exploration is still in infancy (Lan et al., 2019).

A variety of studies have shown that the role of M6A in different tumors is not consistent, and even in the same tumor, the conclusion is opposite. In order to more systematically study the role of M6A in tumors, we have conducted a systematic study on a variety of tumors. We found that in pan-cancer, the expression of m6A methylation regulators is often dysregulated, but presented specific characteristics. Through cluster analysis, we found that there were distinctions in the overall expression characteristics in m6A-related genes in the seven genitourinary cancers and in 11 cancers involving the respiratory, digestive, and head and neck systems. Could this difference provide a perspective for new research? For example, future studies may address the different effects of multiple m6A methylation regulators in multiple human cancers, rather than investigating a single m6A regulator for each tumor as the object of study as in the past. Our findings showed that the expression of m6A-related genes was associated with changes in gene expression in upper digestive system tumors, such as ESCA, STAD, HIHC, and CHOL, which were all up-regulated. At present, there has been no attempt to compare m6A-related genes in different human cancers and their clinical correlations, and these findings will provide a direction for future research. In addition, we found that IGF2BP3 was up-regulated in all 17 tumors investigated, except for THCA, which was basically consistent with that shown by Li et al. (2019).

On univariate Cox regression analysis, we found that in LIHC, with the exception of ZC3H13, higher m6A-related gene expression was associated with worse OS. In the Kaplan–Meier survival curve analysis, we found that the up-regulation of m6A regulators was associated with worse PFI, DFI, DSS, and OS. Among these, the combined high expression of METTL3 and YTHDF1 genes correlated with OS, DSS, DFI, and PFI and indicated worse overall prognosis of patients. Some studies have found that increased gene expression in LIHC may be implicated in the development of cancer. For example, WTAP is significantly up-regulated in LIHC. Through the HuR-ETS1-p21/p27 axis, WTAP contributes to m6A modification contributing to the development of LIHC (Chen Y. et al., 2019). The overexpression of KIAA1429 induces tumor growth and metastasis by inducing the separation of the HuR binding and degradation of GATA3 pre-mRNA (Wang M. et al., 2020). It can also facilitate the migration and invasion of tumor cells by inhibiting ID2 mRNA (Cheng et al., 2019). Through regulation of Snail, a key translator of EMT, METTL3 and YTHDF1 became adverse prognostic factors for OS in patients with LIHC (Lin et al., 2019). Copy number variations (CNV) and DNA methylation were found to be the main causes of aberrant up-regulation of METTL3, which was found to be an independent prognostic factor in the relapse-free survival rate (RFS) and OS rate (Liu G. M. et al., 2020). This report was consistent with our findings on the relationship between up-regulation of METTL3 and YTHDF1 and their association with OS, DSS, DFI, PFI, and worse prognosis of patients. Among the m6A reader genes, the up-regulation of YTHDF1 has been found related to worse prognosis of LIHC (Zhao et al., 2018). YTHDF2 induces proliferation, migration, and colony formation of LIHC cells by promoting METTL3-mediated SOCS2 m6A modification (Chen et al., 2018). In contrast, some studies have shown that YTHDF2 could inhibit the progression of liver cancer by stabilizing EGFR or IL-11 mRNA (Hou et al., 2019; Zhong et al., 2019). These results indicate that the aberrant expression of m6A methylation regulators in LIHC is common, although the controversial results and the potential mechanisms involved are worthy of further study. Nonetheless, these findings provide further evidence of the functional role and potential mechanisms involving a single m6A RNA modification regulation in tumors, although other m6A methylation regulators that may also play a relatively minor role are often ignored. We further analyzed the expression and prognostic significance of multiple genes in LIHC.

We comprehensively analyzed the role and prognostic value of 20 m6A RNA modification regulators in LIHC. According to the consensus clustering of m6A RNA modification regulators, LIHC patients could be stratified into two subgroups in terms of OS. The clustering analysis revealed that all m6A-related genes were highly expressed in the poor prognosis group (cluster2). Univariate analysis indicated that the up-regulated expression of a single gene is related to the poor prognosis of patients, while consensus clustering suggested that there may be some relationship between these genes, which also require further study. Next, we used the LASSO Cox regression algorithm to establish a prognostic gene signature for OS. In accordance with the significant differences in clinical stages and T stages of the high- and low-risk groups, we found that the predictive model constituted by the expression profiles of six genes was a stronger predictor of prognosis of patients in stages I + II than in patients in stages III + IV and was also a better predictor of patients in stages T1 + T2 than those in stages T3 + T4. The risk score results were an independent prognostic factor of LIHC.

Among available studies, we found that those evaluating the combination of m6A-related genes were based on 13 major M6A genes, including METTL3, METTL14, WTAP, KIAA1429, RBM15, ZC3H13, YTHDC1, YTHDC2, YTHDF1, YTHDF2, HNRNPC, FTO, and ALKBH5. In our study, we investigated a total of 20 m6A-related genes that have been identified so far with available data in TCGA. This includes seven genes that have not been previously investigated, including METTL16, RBM15B, YTHDF3, HNRNPA2B1, IGF2BP1, IGF2BP2, and IGF2BP3. In our investigation of the correlation of m6A-related genes, we found that there is a significant relationship between the 20 M6A genes, and in particular, IGF2BP3 and HNRNPA2B1, RBM15B, and YTHDF2. We think these associations may affect the results of single-factor, multi-factor, and cluster analyses and the construction of the prognostic model; thus, the remaining seven genes should not be excluded. In previous studies (Huang et al., 2020a; Liu J. et al., 2020; Wu et al., 2020), the following five genes have been shown to be independent prognostic risk factors, including KIAA1429, METTL3, YTHDF1, YTHDF2, and ZC3H13. Some of them constructed a prognostic model containing five genes. After taking into account the interaction between 20m6A genes, our findings newly showed that IGF2BP3 could be included in the model as an independent risk factor for LIHC, and the risk score resulted to be an independent prognostic factor, which was not found in the previously reported gene combination analysis. This provides a direction for our further experimental research on IGF2BP3 in the future. We also found that the subgroups stratified according to consensus clustering and gene signature were applicable to the early stages I + II and T1 + T2.

The high- and low-risk groups constructed using the m6A methylation regulators stimulated our investigation of the potential mechanisms involved. GSEA analysis showed several significant signaling pathways and cellular processes in the high-risk group were associated with poor prognosis, including processes involving the cell cycle, NER, DNA replication, purine metabolism, pyrimidine metabolism, BER, WNT signal pathway, p53 signaling pathway, and ubiquitin-mediated proteolysis. The cellular processes involved in DNA replication, purine metabolism, BER, and NER were consistent with previous reports, indicating DNA damage was regulated by m6A-induced methylation (Xiang et al., 2017). The response of m6A in DNA damage is transient. This modification is regulated by N6-methyltransferase-like protein 3 (METTL3) and obesity-associated protein (FTO) and is mostly found in poly(A) transcripts. Previous studies have shown that M6A plays an important role in the DNA damage response, because the lack of METTL3 leads to delayed cell repair and increased sensitivity to ultraviolet light (Xiang et al., 2017). Several biological processes related to malignant tumors are noteworthy, including regulation of cell cycle, WNT signaling pathway, p53 signaling pathway, and ubiquitin-mediated proteolysis. It has been reported that the RNA METTL3 and miR-186 regulate hepatoblastoma progression through the Wnt/β-catenin signaling pathway (Cui et al., 2020). Overall, this evidence indicated that the characteristic expression of m6A methylation regulators has great prospect as prognostic indicators of LIHC. These genes and their relative pathways may be latent treatment objectives for LIHC.

However, our research still has some limitations. First of all, most of the LIHC patients we studied were white and Asian. However, it is not clear about the geographical location of the specific life of these Asians, so whether the final results of the study are also applicable to the Chinese population needs to be further verified by clinical sampling in Chinese patients. Second, the sample size may be insufficient because many cases lacked clinical information. Third, several significant prognostic factors for LIHC were not evaluated in the study, such as hepatitis B virus infection and abnormal liver function, which may lead to changes in the correlation between m6A-related genes and prognosis. Fourth, the description of the potential mechanism of the role of these up-regulated genes in prognosis was not based on experimental evidence; thus, further confirmation is required in the future.

## Conclusion

Based on an integrated bioinformatics analysis, the present study identified several genes associated with m6A in LIHC. In particular, METTL3 and YTHDF1 expression were found to be correlated with an increased risk and were included in an m6A-related gene signature for predicting prognosis of LIHC. The poor prognosis group was closely associated with a poor response to DNA damage repair and several biological processes associated with malignant tumors. In the pan-cancer analysis used in our preliminary study, we found that changes in m6A-related genes occurred across different cancer types. This provides an important rationale to guide future cross-cancer studies. The mechanisms indicated for the role played by up-regulated genes were only theoretical; thus, functional experiments and prospective clinical studies are needed to validate our findings.

## Data Availability Statement

The original contributions presented in the study are included in the article/supplementary material, further inquiries can be directed to the corresponding author/s.

## Author Contributions

L-WQ and J-MH designed the study. L-WQ and C-HJ collected the literature. L-WQ, J-HJ, and J-MH performed statistical analyses. L-WQ, J-HJ, J-MH, and C-HJ analyzed the data. L-WQ wrote the manuscript. All authors listedhave made substantial, direct and intellectual contribution to the work and approved it for publication.

## Conflict of Interest

The authors declare that the research was conducted in the absence of any commercial or financial relationships that could be construed as a potential conflict of interest.

## Footnotes

1 https://xena.ucsc.edu/

2 https://portal.gdc.cancer.gov/

3 http://www.string-db.org/

4 https://www.r-project.org/
